# Virtual reality as a potential therapy in a rehabilitation sanatorium for patients after ischemic stroke: impact on quality of life and social participation—a randomized trial

**DOI:** 10.3389/fresc.2025.1539175

**Published:** 2025-06-02

**Authors:** Marcela Dabrowská, Lucie Honzíková, Dalibor Pastucha, Miroslav Janura, Hana Tomášková, Iva Fiedorová, Šárka A. Čechová, Jana Trdá, Milan Elfmark

**Affiliations:** ^1^ Department of Rehabilitation and Sports Medicine, Faculty of Medicine, University of Ostrava, Ostrava, Czechia; ^2^ Department of Rehabilitation and Sports Medicine, University Hospital Ostrava, Ostrava, Czechia; ^3^ Department of Natural Sciences in Kinanthropology, Faculty of Physical Culture, Palacký University in Olomouc, Olomouc, Czechia; ^4^ Department of Epidemiology and Public Health, Faculty of Medicine, University of Ostrava, Ostrava, Czechia; ^5^ VR LIFE s.r.o. Ostrava, Ostrava, Czechia

**Keywords:** activities of daily living, cognitive functions, ischemic stroke, physiotherapy, self-sufficiency, standardized questionnaires

## Abstract

**Objective:**

The aim of this study was to determine whether adding virtual reality therapy to conventional rehabilitation improves the quality of life, cognitive functions, and social participation of patients after an ischemic stroke.

**Design:**

Randomized controlled study conducted in a rehabilitation center.

**Participants:**

The experimental group with therapy in virtual reality included 25 patients (age 59.4 ± 8.9 years), and the control group with conventional therapy consisted of 25 patients (age 63.0 ± 8.8 years). Inclusion criteria for the study were: age 40–79 years, stable condition, Mini-Mental State Examination >25 points, intact vision, preserved grip function of the thumb and index finger of the affected limb, functional mobility according to the functional ambulatory category (FAC) 3–5, and no other neurological disease.

**Methods:**

The Mini Mental State Examination, the Barthel Index, the Extended Barthel Index, and the WHO Disability Assessment Schedule 2.0 were used to assess cognitive function, quality of life, and self-sufficiency. Based on the results of normality test were used: *t*-test for two samples (age, time since stroke), the chi-square test (gender), nonparametric paired Wilcoxon test and Mann–Whitney *U* test. Friedman analysis was used to analyze repeated measures and a *post hoc* test Scheffe test was used to compare differences. Statistical tests were evaluated at the 5% significance level.

**Results:**

No significant differences were found between the experimental and control groups in any of the tests applied after treatment. Significant differences emerged after treatment in all WHODAS domains studied for each group compared to the measurement before therapy. In the experimental group, the positive effects of therapy persisted 1 year after the end of therapy compared to the measurement before therapy.

**Conclusions:**

Virtual reality has proven to be a suitable adjunct to conventional therapy for post stroke patients and offers an advantage over traditional rehabilitation methods in that it allows training in activities of daily living that are not commonly available in a hospital setting.

## 1 Introduction

Despite significant transformation in treatment and diagnosis, stroke is one of the most common acute conditions in neurology (1). The consequences of stroke are associated with severe disability (2), functional impairment, cognitive deficits and high mortality (3). Impairments in memory, attention, decision-making, orientation, and other cognitive functions significantly affect social life, limiting inclusion in society and participation in social activities. These changes can complicate communication and interaction with others and significantly impair quality of life (4). Receptive and expressive communication are key to human interaction, and their training can enhance cognitive functions such as verbal memory and communication skills. Furthermore, practicing language skills and the ability to interpret and express ideas can strengthen cognitive functions such as the ability to process information, express fluency and interact effectively with the environment. After a stroke, returning to a normal level of communication and participation in social life can be highly individual. It depends on the severity of the disability, the quality of rehabilitation, and the support of the environment. The variability of dysfunctions and their development significantly affect self-sufficiency and quality of life, and are closely related to the satisfaction of human needs, complicating the return to normal daily life (5).

The increasing number of patients with severe sequelae after a stroke is negatively affecting their quality of life and that of their families and increasing costs to the health and social care system. These patients often require intensive care and support. Families of patients face emotional stress and financial pressure as they are forced to provide care, adapt their homes, and dedicate more time to care for their loved ones (5, 6). Studies report that up to 57% of patients need help with personal ADL (Activities of Daily Living) corresponding to needs such as personal hygiene, bathing, dressing, self-feeding, toilet use, and controlling urination and defecation (7). Patients with severe deficits who cannot work depend on welfare benefits, increasing social inequalities and reducing economic productivity.

Coordinated rehabilitation is the key to maximize recovery from function and improve quality of life for patients with various types of functional impairments and cognitive deficits (8). It should be individualized and tailored to the specific needs and goals of each patient. In addition to physical and cognitive aspects, social and emotional rehabilitation is also important. In patients after stroke, neurorehabilitation is applied primarily with the main objective of quantitative and qualitative improvement in daily activities. This enables the person with neurological impairment to achieve optimal levels of physical, cognitive, emotional, communicative, social, occupational, and functional activity and leads to improved quality of life (5, 9). Neurorehabilitation may also include psychotherapy or psychosocial support, with the aim of improving the emotional and psychological health of patients who may be struggling with depression, anxiety, or other psychological difficulties. Effective rehabilitation programs aimed at promoting and developing motor and cognitive function and independence are essential to improve functional ability and quality of life for patients recovering from stroke (10). Virtual reality-augmented rehabilitation can contribute to this.

Virtual reality (VR) is a sophisticated technological tool with many potential benefits used in neurorehabilitation. Virtual reality therapy has become widespread in recent years and is increasingly becoming an integral part of the treatment of motor and cognitive dysfunctions in post-stroke patients (10). Features such as immersion, imagery, and interaction play a key role in motivating patients to increase repetition of targeted movement activities and enhancing the effectiveness of therapy (11). VR helps in rehabilitation because it allows patients to visualize the movements and activities they would like to perform, in a safe and controlled environment. Through multisensory stimulation, different senses are engaged, including vision and hearing, which also improves and promotes motor learning. With an increase in the number of exercises performed, not only is the ability to move the affected limb improved, but more active participation and participation in society, thus improving quality of life (10, 12). Conventional therapy is mostly concerned with routine care and patients may lose motivation and adherence to treatment as therapeutic movements become tedious and monotonous over time (13). Available studies report that virtual reality training is motivating, enjoyable, and more engaging than conventional therapeutic exercises (13, 14). Virtual games, competitions, rewards, but also the possibility to change the environment, create different scenarios and tasks that can be adapted to the individual needs of patients, make VR therapy an effective technology. Meta-analyses and systematic reviews, such as those by Laver et al. (10) and Zhang et al. (15), confirm the effectiveness of VR in post-stroke rehabilitation, with a focus on improving motor and cognitive function. However, studies examining the long-term effects of VR on patients' participation, self-sufficiency, and overall quality of life are still scarce and this area requires further research (16). Hao et al. (17) found that VR therapy can improve interhemispheric balance, cortical connectivity, and cortical mapping of affected muscles in post-stroke patients. Therefore, the objective of the present study was to test whether adding VR therapy to conventional rehabilitation will improve quality of life, cognitive function, and participation in social life in patients after ischemic stroke.

## 2 Material and methods

This randomized controlled trial with a parallel group included patients admitted to a rehabilitation sanatorium. This sanatorium specializes in complex neurorehabilitation in all phases of the disease—acute, subacute and chronic—after a first ischemic stroke in the middle cerebral artery. The study was approved by the Ethics Committee of the Faculty of Medicine of the University of Ostrava (12/2022) and all participants provided informed consent.

### 2.1 Participants

The study included patients within 6 months of a first ischemic stroke in the media of a. cerebri who were referred for rehabilitation of 4–5 weeks in a rehabilitation sanatorium.

Inclusion criteria for the study were age 40–79 years, stable condition, Mini-Mental State Examination above 25 points, intact vision, preserved grip function of the thumb and index finger of the affected limb, and functional mobility according to the functional ambulatory category (FAC) 3–5 (15). Study exclusion criteria: age >40 a <79 decompensated state, cardiovascular instability, severe fatal and severe cognitive impairment, low functional mobility according to FAC 0–2, dementia with mini-mental state examination >24 points, severe visual impairment, personal history of epilepsy.

**Figure 1:**
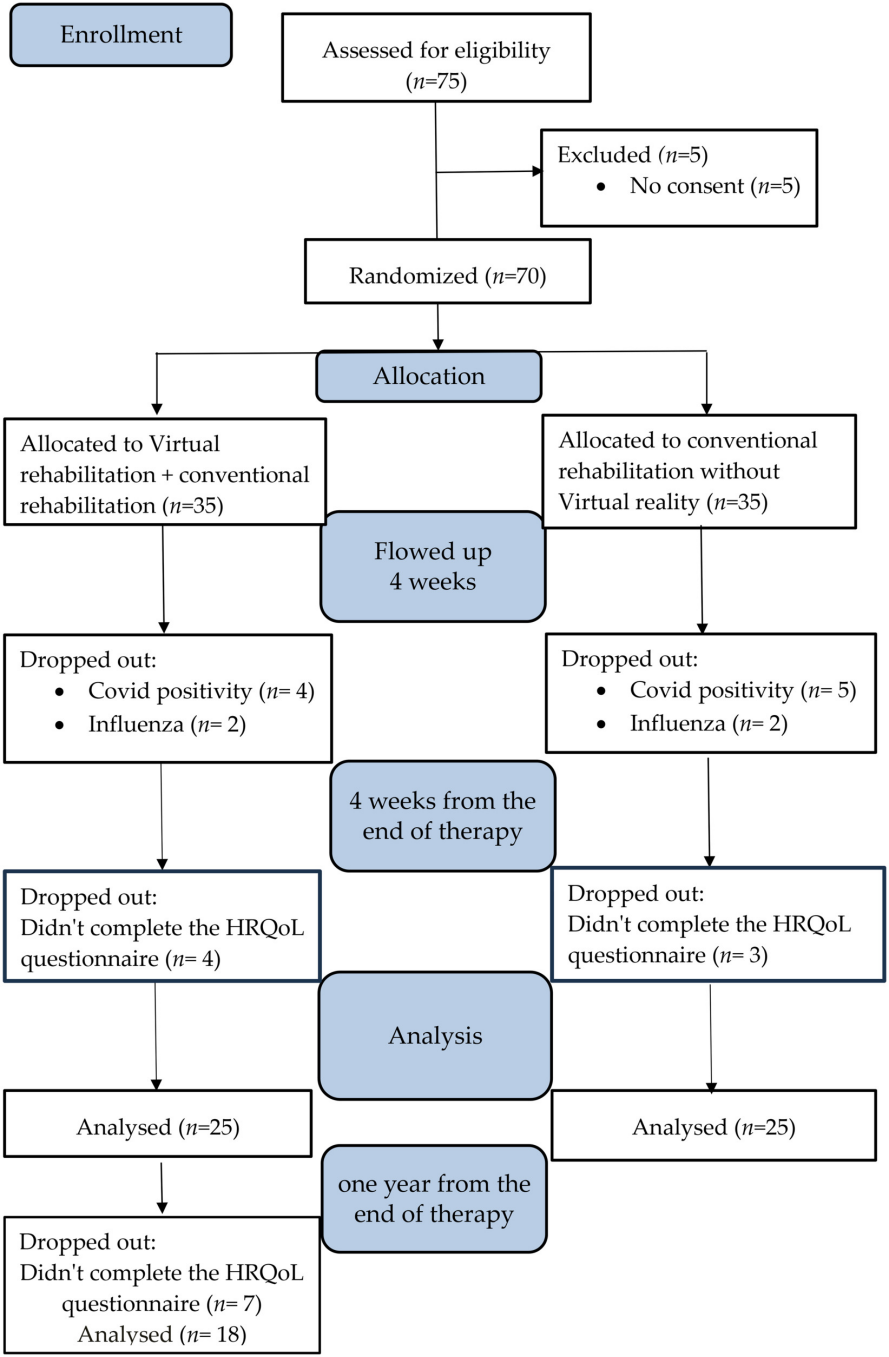
The diagram shows and justifies the changes in the numbers of patients in each group over the course of the study.

The selected patients had no other neurological disease that could affect the measurement results. Patients were randomly divided into two groups according to the type of therapy (Figure 1): experimental (conventional combined with VR) and control (conventional).

### 2.2 Measurement procedures

The Mini-Mental State Examination, the Barthel Index, the Barthel Extended Test, and WHODAS 2.0 were used to assess cognitive function, quality of life, and self-sufficiency.

The Mini Mental State Examination (MMSE) is the most widely used 10-item test to detect cognitive deficits, evaluate orientation, attention, numeracy, memory, speech, and construction skills (16, 18). The MMSE does not assess impairment in executive function but does a good job of differentiating moderate dementia from normal aging. The maximum score is 30 points, with a score of <24 points starting the dementia band (18).

The Barthel Index (BI) is a tool that assesses functional ability in basic activities of daily living post-stroke (16, 19). The BI assesses 10 activities of daily living, such as eating, bathing, personal hygiene, dressing, continence and toilet use, transferring, mobility, and stair walking. Activities can be scored from 0 to 15 points. The total score range is 0–100 points (19, 20). A total score of 0–20 points indicates very severe dysfunction, a score of 25–45 points severe dysfunction, a score of 50–70 points moderate functional impairment, a score of 75–95 points mild functional impairment, and a score of 100 points full ADL self-care (21).

The Extended Barthel Index (EBI) helps assess the need for assistance in activities of daily living (ADLs) that require certain cognitive abilities (22, 23). The test consists of 6 cognitive elements that assess comprehension, communication, social interaction, daily problem solving, memory, learning and orientation, vision, and neglect syndrome. Each item is scored on a 0–5 scale (0—worst performance, 5—the best performance), with a maximum of 15 points per item. The maximum score is 90 points. The extended Barthel test classifies patients into three categories: severe cognitive deficit (0–15), moderate cognitive limitation (20–65), no or mild cognitive deficit (70–90) (21–23).

The WHODAS 2.0 is an international standardized generic test with excellent psychometric properties developed for stroke patients that assesses disability as defined by the International Classification of Functional Abilities. The WHODAS 2.0 includes six core domains of quality of life. Domain 1 assesses understanding and communication, domain 2 mobility, domain 3 self-care, domains 4 and 5 include relationships with people and assess activities of living, and domain 6 focuses on participation in society. Domain scores and total score can be calculated using the SPSS (Statistical Package for the Social Sciences) algorithm. The item response theory is applied in the scoring, where individual items may have different weights. Disability is expressed as a percentage, from 0% to 100%. The lower the score, the better the outcome (quality of life) (21, 24).

### 2.3 Procedure

All patients underwent an initial examination on the first day after admission to the sanatorium, which was performed by an experienced occupational therapist in a quiet environment without distractions. The examination included the MMSE, BI, EBI, WHODAS 2.0. All VR therapies were followed by exit examination, which included the MMSE, BI, EBI, and WHODAS 2.0. After 4 weeks from the end of therapy, a final evaluation was performed using the WHODAS 2.0 questionnaire. One year after the end of the therapy, the experimental group was evaluated with the WHODAS 2.0 questionnaire.

### 2.4 Instruments used

The Meta Quest 2 headset (Meta Platforms, New York, NY, USA) was used for the virtual reality application. The device has 6 GB of RAM with a Qualcomm Snapdragon XR2 platform. It consists of a projection helmet that houses two eye screens with focus adjustment, a goggle attachment, and two controllers. The low-transparency backlight LCD display with a resolution of 1,832 × 1,920 pixels per eye provides visual fidelity of approximately 21 pixels per degree without blurring or double artifacts. Adjustable straps are used to secure the helmet to the head. Both touch controllers have multiple control buttons, a joystick, and an adjustable fixation strap that attaches to the wrist to prevent the controller from possibly falling out of the hand. The built-in Bluetooth and an app on a mobile phone or tablet allow the therapist to monitor what the patient sees while analyzing how effectively they perform each task.

### 2.5 Intervention

In addition to conventional therapy, the patients enrolled in the experimental group also received training using the VR device Oculus Quest 2, software 0.4.1. In total, patients underwent a minimum of 10 and a maximum of 15 20-min VR sessions, three times a week for 4–5 weeks. The duration of 20 min was based on the time the patient spent doing actual exercise with a physiotherapist or occupational therapist. A standard therapy unit lasts 30 min, but this time does not include the interview with the therapist, assessment of the condition, and preparation for exercise. The initial three therapy sessions, which lasted a maximum of 10 min, focused on familiarization with the functions to control the system. First steps for Quest 2, developed by Meta Platforms, New York, NY, USA, were used for therapy. From the fourth therapy, the VITALIS Pro VR software was used in the intervention. The patients were seated for the first two therapies and completed the third and subsequent therapies standing. All patients completed the exercises in a total of 4 programs: free painting, 2D tracing, 3D painting, and puzzle. In the “free painting” program, they had to draw specific shapes, in 2D and 3D they traced the shapes shown. The “puzzle” program had three levels of difficulty and a hint option. The patients performed each task in three types of environment, namely, forest, space, and sea. Each environment also conveyed different sound sensations to the patients. When performing tasks in the forest environment, the patients heard birds singing, in the sea environment it was the sound of waves, and in the space environment it was computer music. A therapist was present in the room throughout the exercise to monitor the patient for symptoms indicating sudden instability, seizures, shoulder, arm, or hand pain. If the patient did not feel well at any time during the exercise, the therapist instructed to stop the task. Within conventional therapy, patients were indicated individual physical therapy on a neurophysiological basis twice a week for 30 min. The next part of the therapy focused on occupational therapy aimed at developing self-sufficiency, which included targeted training in basic activities of daily living and instrumental activities of daily living. In addition, the therapy focused on improving gross and fine motor skills, monomanual and bimanual activities and motor coordination. This concept is based on activity-based rehabilitation or task-oriented training that emphasizes the development of practical skills to increase the patient's functional independence and ability to return to daily life. Occupational therapy was performed twice a week for 30 min. In addition, patients underwent an iodobromine bath + wrap, oxygen therapy, whirlpool bath, pool exercise, dry hot compresses, classical massage, mechanotherapy, CO2 gas injections, four-chamber bath and electrical stimulation twice a week.

Patients in the control group were indicated individual physical therapy on a neurophysiological basis twice a week for 30 min. The next part of the therapy focused on occupational therapy, that was performed twice a week for 30 min. In addition, patients underwent an iodobromine bath + wrap, oxygen therapy, whirlpool bath, pool exercise, dry hot compresses, classical massage, mechanotherapy, CO2 gas injections, four-chamber bath and electrical stimulation twice a week.

### 2.6 Statistical analysis

For statistical processing of data, Stata version 17 was used. The normality of data was tested by the Shapiro–Wilk test. Based on the results of normality test were used: *t*-test for two samples (age, time since stroke), the chi-square test (gender), nonparametric paired Wilcoxon test and Mann–Whitney *U* test. Friedman analysis was used to analyze repeated measures and a *post hoc* test using the Scheffe test was used to compare differences. Statistical tests were evaluated at the 5% significance level. The coefficient *η*^2^ was used to determine effect size, where a value of 0.01, 0.06, and 0.14 represents a small, medium, and large effect (25).

## 3 Results

A flow chart of the process of patient selection for the study is shown in Figure 1. A total of 75 patients were included for randomization, 50 of whom met the entry and exit criteria, completed follow-up tests and completed the WHODAS 2 questionnaire 4 weeks after the end of therapy. Twenty-five patients were excluded from the study for premature termination of their stay in the rehabilitation sanatorium due to illness, positive for COVID 19, or failure to complete the quality of life questionnaire 4 weeks after the end of therapy.

The sociodemographic and clinical characteristics of the patients are shown in Table 1. There were no statistically significant differences between the two groups in age, side of the lesion and time since stroke. The experimental group included 25 patients (mean age 59.4 ± 8.9 years; 12 women, 13 men), 15 of whom were employed before the stroke and 10 of whom were receiving a retirement pension. The control group also consisted of 25 patients (mean age 63.0 ± 8.8 years; 12 women, 13 men), 12 of whom were employed before stroke and 13 of whom received a retirement pension.

**Table 1:** Basic sociodemographic characteristics of the experimental and control groups and their comparison.

Variable	Experimental group (*n* = 25)	Control group (*n* = 25)	*p* (Experimental vs. control)
Age^a^	59.4 ± 8.9	63.0 ± 8.8	0.158
Side of the lesion (right/left) *n*, (%)^b^	17 (68)/8 (32)	14 (56)/11 (44)	0.561
Time since stroke (days)^a^	111.2 ± 44.6	117.4 ± 34.1	0.762

^a^ Variable had a normal distribution, values are presented as mean ± standard deviation; two-sample *t*-test was used to compare groups.

^b^ Variable did not have a normal distribution: chi-square test was used.

Table 2 shows the comparison of MMSE, BI and EBI test results before and after therapy and the differences between the two groups. After the therapy, statistically significant improvements in the scores of the individual tests were found in both groups compared to the values before therapy. No significant differences were found between the experimental and control groups in any of the tests applied before and after therapy (*p* > 0.05).

**Table 2:** Scores of MMSE, BI, EBI of both groups and their comparison.

Variable	Experimental group (*n* = 25)			Control group (*n* = 25)		
	M1	M2	*p*	M1	M2	*p*
MMSE^a^	27.6 ± 2.1	28.2 ± 1.8	<0.001	27.0 ± 1.3	27.6 ± 1.5	<0.001
BI^b^	90 (75; 100)	100 (90; 100)	<0.001	95 (85; 98)	95 (95; 100)	<0.001
EBI^b^	90 (75; 90)	90 (85; 90)	0.004	90 (85; 90)	90 (85; 90)	0.008

M1 = measurement before therapy, M2 = measurement after therapy.

^a^ Variable had a normal distribution, values are presented as mean ± standard deviation; one-sample *t*-test was used to compare measurements and two-sample *t*-test to compare groups.

^b^ Variable did not have a normal distribution, values are presented as median, first and third quartile; Wilcoxon test was used to compare measurements and Mann–Whitney *U* test to compare groups.

Table 3 shows total score of the quality of life assessment using the WHODAS 2 questionnaire in both groups and Table 4 describes the scores of the individual domains of the WHODAS 2 for each measure in both groups. In both groups, there was a significant improvement (reduction) in the WHODAS 2.0 questionnaire total score between measurements after therapy and 4-week after therapy compared to measurements before therapy. The same results were found for the individual domains of the WHODAS 2.0 questionnaire in the groups. In the experimental group, a significant improvement (reduction) in the total score and in the individual domains of the WHODAS 2.0 questionnaire was also found 1 year after the end of therapy compared to the measure before therapy (*p* < 0.05).

**Table 3:** Scores of WHODAS 2 of both groups and their comparison.

Variable	Experimental (*n* = 25)		Control (*n* = 25)		ANOVA		
	M	SD	M	SD	*F ratio*	*df*	*η* ^2^
WHODAS							
Measurement 1	38.5	22.0	30.6	16.8	0.93	1.48	0.02
Measurement 2	29.4	19.4	25.1	14.9	99.02**	2.96	0.67
Measurement 3	26.0	17.8	24.0	14.6	8.61**	2.96	0.15

Measurement 1 = measurement before therapy, Measurement 2 = measurement after therapy, Measurement 3 = measurement 4 weeks after therapy, M, mean; SD, standard deviation.

*Statistical significance *p* < 0.05.

** *p* < 0.01, *η*^2^—effect size.

**Table 4:** Scores of WHODAS 2 domains of both groups and their comparison.

Variable	Experimental (*n* = 25)		Control (*n* = 25)		ANOVA		
	M	SD	M	SD	*F ratio*	*df*	*η* ^2^
Cognitive							
Measurement 1	34.2	29.6	26.0	22.7	0.44	1.48	0.01
Measurement 2	24.7	22.6	21.0	19.3	22.97**	2.96	0.32
Measurement 3	22.6	21.5	19.3	18.0	0.51	2.96	0.01
Mobility							
Measurement 1	44.0	26.8	34.0	20.1	1.09	1.48	0.02
Measurement 2	32.4	23.5	26.0	17.0	60.44**	2.96	0.56
Measurement 3	27.2	23.0	25.0	17.3	5.08**	2.96	0.10
ADL							
Measurement 1	38.0	29.4	29.6	19.6	0.69	1.48	0.01
Measurement 2	26.8	22.8	21.8	17.3	42.78**	2.96	0.47
Measurement 3	22.0	21.2	20.8	17.1	3.26*	2.96	0.06
Relations							
Measurement 1	29.4	20.0	24.8	14.9	0.15	1.48	0.00
Measurement 2	23.4	16.4	22.6	13.9	20.36**	2.96	0.30
Measurement 3	20.8	15.4	21.2	12.9	3.59*	2.96	0.07
Life activity							
Measurement 1	30.4	18.6	24.5	14.9	1.13	1.48	0.02
Measurement 2	26.3	17.1	21.8	13.0	20.11**	2.96	0.30
Measurement 3	23.9	16.4	20.6	11.7	1.24*	2.96	0.03
Participation							
Measurement 1	53.7	21.2	43.5	18.0	1.70	1.48	0.03
Measurement 2	44.3	21.9	37.2	16.6	28.31**	2.96	0.37
Measurement 3	40.3	20.3	37.0	17.5	3.11	2.96	0.06

Measurement 1 = measurement before therapy, Measurement 2 = measurement after therapy, Measurement 3 = measurement 4 weeks after therapy, M, mean; SD, standard deviation.

* *p* < 0.05.

** *p* < 0.01, *η*^2^—effect size.

## 4 Discussion

Published studies suggest that VR therapy provides benefits such as improved mobility, functional independence, and quality of life for patients (26, 27). On the contrary, other studies have reported that VR therapy provides only modest improvements over traditional methods (28), and that VR therapy alone is not more effective than conventional approaches in improving upper limb function, ADLs, and quality of life (10). Zhang et al. (29) published that VR-based therapies are effective in improving executive function, memory, and visuospatial function in post-stroke patients. Patients included in our study did not have significant cognitive deficits, yet both groups showed significant improvement in this area after therapy. The patients in the experimental group showed significant improvement after VR therapy in all areas assessed by the quality of life questionnaire, cognitive tests, and self-sufficiency. The positive effect persisted 4 weeks after therapy and a follow-up assessment at 1 year found a significant improvement compared to measurement before therapy. Patients in the control group also perceived a significant improvement in quality of life after therapy, which they expressed in a questionnaire. However, no statistically significant improvement was observed when comparing the results of the tests with the experimental group after the end of therapy and 4 weeks after the end of therapy.

Zielina et al. (9) and Laver et al. (10) report that VR can significantly contribute to improved motor skills, with increased patient motivation playing a key role in making patients more actively engaged in rehabilitation (5, 9). VR programs are designed to simulate real-life situations and interactions (30, 31), thus naturally promoting intrinsic motivation in patients (10, 19, 32). The addition of gaming elements further increases engagement levels and fosters a sense of personal accomplishment (26, 27). Our patients reported that the game environment and the desire to complete the task motivated them to perform even activities that they would otherwise perceive as monotonous or uninteresting. This motivational factor was essential not only for regular engagement in exercise, but also for achieving the required number of repetitions of movements, which contributed to promoting neuroplasticity and subsequent better ADLs and participation in routine activities. Also, Perez-Marcos (11) states that the use of VR stimulates neuroplasticity, leading to more efficient recovery and better adaptation of the brain to new movement patterns. Moreover, Broeren et al. (14) demonstrated that the combination of VR and haptic feedback yields positive results in motor rehabilitation, making VR a valuable adjunct to conventional therapy.

The cognitive benefits of VR therapy extend beyond motor recovery, engaging key processes such as attention, memory, and executive function (29). Patients in our study, despite not having significant cognitive deficits, often reported difficulties with memory and learning new skills. These challenges impacted their confidence and overall independence in daily life. However, VR therapy provided a structured and engaging environment that facilitated cognitive stimulation, leading to subjective and measurable improvements in cognitive performance and self-sufficiency. Research shows that regular VR training promotes not only motor function but also cognitive flexibility and adaptability to new situations (30, 31). Zhang et al. (29) demonstrated that VR-based interventions effectively improve executive function, memory, and visuospatial abilities in post-stroke patients. Our findings are consistent with these results, as patients in the experimental group showed statistically significant improvements in cognitive function, quality of life, and self-sufficiency scores. These benefits persisted in the long term. While VR can replicate real-world scenarios and enhance motor-cognitive integration, it does not replace a comprehensive rehabilitation program involving a multidisciplinary team (29). The long-term sustainability of VR therapy effects remains a subject of ongoing research, as robust longitudinal studies evaluating its durability are still limited (30).

Studies by Glymour et al. (32) and Hu et al. (33) suggest that continuous social support and strong interpersonal connections are crucial for cognitive recovery and overall quality of life in post-stroke patients. Our findings support these claims because patients who returned to a home environment with adequate family and social support reported higher levels of subjective satisfaction and independence. This trend is consistent with the findings of Chang et al. (34) and Lee et al. (35), who also described the positive impact of social integration on health status and quality of life post-stroke.

Studies suggest that social support positively influences recovery, but the results depend on the extent of disability (36). The duration and consistency of social support is crucial, as continuous support and social interaction have a long-term positive effect on recovery (32). The quality and nature of social support also play an important role. Emotional and instrumental support in activities of daily living is essential for improving quality of life. In general, social support and strong social ties provide patients with emotional encouragement and motivation. It is essential that this support is tailored to the individual needs and circumstances of each patient. Patients involved in our study rated engagement in social activities as difficult to extremely difficult. This limitation may lead to prolonged social isolation, either at home or in institutional care, which negatively affects psychological well-being and may contribute to depression (36). Hu et al. (33) and Lee et al. (35) concluded that cognition levels, mobility, and WHODAS 2.0 total scores are strong predictors of institutionalization. In our study, most patients were in a relationship and all planned to return to their home environment. This factor is crucial, as quality social support, particularly from close family members, plays a key role in improving quality of life and cognitive recovery. Patients with strong social bonds perceive greater emotional support and motivation, facilitating their reintegration into daily life. Returning home and maintaining family relationships also fosters a sense of self-sufficiency and reduces the risk of social isolation. Previous studies have demonstrated that the ability to resume one's former lifestyle, including participation in social and domestic activities, is a significant determinant of patient satisfaction and perceived quality of life (38, 39). Despite these advantages, various social barriers and prejudices related to disability still limit opportunities for social participation. It is therefore essential to create conditions and environments that enable patients with disabilities to participate fully in social activities and integrate into society. This includes physical environmental adaptations, educational support and mental health assistance. As suggested by Broeren et al. (14) and Zhang et al. (29), VR therapy can play an important role in bridging these gaps by simulating real-world scenarios and facilitating cognitive-motor training in an engaging and accessible way. Our findings suggest that VR therapy promotes motivation and cognitive stimulation, which may help to reduce social isolation and improve overall quality of life.

In the assessment of interpersonal relationships, our patients reported persistent mild to moderate difficulties in interacting with strangers and forming new friendships. These challenges may stem from cognitive deficits, such as impaired ability to read non-verbal cues, interpret emotions, and respond appropriately to social situations (7). Research suggests that these deficits are closely linked to post-stroke cognitive impairments affecting social cognition and executive functions, which in turn influence social integration (36). As a result, patients may struggle to build trust and resolve conflicts, further complicating their social interactions. Studies indicate that untreated or neglected deficits in these areas often lead to social isolation and loneliness, negatively impacting psychological well-being and overall quality of life (33). Women, in particular, were more likely to report emotional difficulties related to their condition, including persistent problems with sexual activity even after rehabilitation (36). Motor deficits, combined with depression, anxiety, and emotional distress, can significantly affect sexual desire and performance, as suggested by research on the psychological impact of stroke on self-rated health (7). Additionally, a lack of open communication between patients and their partners about these issues can lead to misunderstandings, increased stress, and further reductions in quality of life (37).

Living with a disability affects a person's ability to participate actively in the workforce and in economic and social life (35, 38). Many post-stroke patients experience permanent disabilities that prevent them from returning to their original employment. The loss of the ability to work or the increased costs of care, long-term rehabilitation, medication, compensatory aids, or social services have significant economic impacts on individuals and their families. In our study, 27 patients were employed before their stroke and all reported great difficulty with their daily work. In general, the self-employed, people in managerial or administrative positions show a higher frequency of return to work than those with less education. This may be because these positions can provide greater flexibility or the ability to work from home, which is advantageous for post-stroke patients. Of the 27 patients, only nine respondents planned to return to work within 6 months after completing rehabilitation.

Changes in health status and the extent of disability often require patients to allocate more resources to their care. In cases where sufficient funds are not available, patients are not only unable to meet their basic needs but may also face significant financial difficulties. In our study, patients reported moderate difficulties due to the financial burden caused by their health problems, which consumed their own and their family's financial resources. Many patients reported that their health problems significantly deplete their financial reserves. It is essential for stroke patients to have access to appropriate financial and social support to enable them to manage the costs of their healthcare and also to facilitate their lifestyle adjustment. This may include strategies for returning to work or adjusting their professional role. Lee et al. (35) found in their study that the perception of social support was positively correlated with recovery motivation. Patients who felt supported socially showed stronger motivation to recover, which in turn was associated with lower levels of disability. In a study by Chang (39), 60% of patients under 65 years of age returned to work within 6 months of having a stroke. These individuals demonstrated better emotional well-being compared to those who were unable to return to work.

Like any serious chronic illness, stroke undoubtedly reduces the quality of life of an individual because it changes his or her life situation. The patient's response to the new situation depends on many factors. Mobility and walking are among the important human activities that allow us to be productive and actively participate in life among family or community members (40). Difficult mobility will limit patients' reintegration into society (41, 42). VR programs may have some advantages over conventional rehabilitation approaches because they provide opportunities to practice activities of daily living, improve mobility, and learn new skills that cannot or cannot be practiced in a hospital setting (10, 43).

The use of virtual reality in the rehabilitation of stroke patients offers a number of advantages, such as improved motor and cognitive function, self-sufficiency, coordination, and balance. VR is an attractive rehabilitation tool because it motivates patients and increases their involvement in long-term treatment. However, to truly be effective for VR programs for post-stroke patients, more research is needed on the optimal selection and timing of these technologies in the treatment process.

## 5 Limits of the study

One limitation of the study was that some patients found virtual reality tasks too easy after a certain amount of time in therapy and expressed interest in more challenging tasks. The validity of the results may also have been affected by the small sample size, which was further reduced due to the patients' illnesses and cases of COVID-19 infection. Another limiting factor in the study is the presence of comorbidities in the patients, which may have affected the effect of the therapy. Furthermore, the uneven distribution of these comorbidities between the experimental and control groups may have compromised the comparability of the results and reduced the overall validity of the study.

## 6 Conclusion

The results of this study show that the inclusion of virtual reality as an adjunct to standard therapy in patients with stroke did not result in significantly different changes in quality of life, cognitive function, and social participation compared to patients who underwent conventional therapy alone. Virtual reality has proven to be a motivating tool for patients and provides an advantage over traditional rehabilitation methods in terms of the ability to practice activities of daily living and learn new skills that are not commonly available in a hospital setting.

## Data Availability

The raw data supporting the conclusions of this article will be made available by the authors, without undue reservation.
